# Acid ceramidase modulates the lipid profile and exacerbates sensitivity to ferroptosis in senescent cells

**DOI:** 10.21203/rs.3.rs-8117957/v1

**Published:** 2025-11-28

**Authors:** David Soriano-Castell, Marie Goujon, Nawab John Dar, Antonio Currais, Pamela Maher

**Affiliations:** 1 Cellular Neurobiology Laboratory, The Salk Institute for Biological Studies, 10010 N. Torrey Pines Rd. La Jolla, CA 92037, USA.; 2 MedInUP – Center for Drug Discovery and Innovative Medicines, Department of Biomedicine, Faculty of Medicine, University of Porto, 4200-319, Porto, Portugal

## Abstract

Cellular senescence, a complex biological process characterized by irreversible cell cycle arrest and the senescence-associated secretory phenotype, has emerged as a critical target for therapeutic development for age-related diseases. Ferroptosis, an iron-dependent regulated cell death pathway driven by the accumulation of lipid peroxidation in cell membranes, has been implicated in neurodegenerative diseases and other age-related disorders. This study investigated the relationship between cellular senescence and ferroptosis. Using human fetal lung Wi-38 fibroblasts induced to senesce via replicative exhaustion, we report a novel role for acid ceramidase (ACase), which breaks down ceramides into sphingosine and free fatty acids, in regulating the sensitivity of senescent cells to lipid peroxidation and ferroptosis through the modulation of polyunsaturated fatty acid composition of membrane phospholipids. Furthermore, we demonstrate a cell non-autonomous paracrine sensitization of non-senescent cells to ferroptosis by senescent cells. Together, these findings unveil ACase as a novel regulator of the ferroptosis pathway and open promising therapeutic avenues for targeting senescence-linked disorders and advancing healthy aging strategies.

## Introduction

Although emerging evidence suggests a potential link between cellular senescence and the regulated cell death pathway of ferroptosis, the molecular mechanisms underpinning this interplay remain largely unresolved^1,2^. Ferroptosis is a non-apoptotic iron-dependent cell death pathway, also called oxytosis^3,4^, that can be induced by glutathione (GSH) depletion or the inhibition of the GSH-dependent peroxidase GPx4. These processes generate a burst of reactive oxygen species (ROS), resulting in increased peroxidation of polyunsaturated fatty acids (PUFAs) in cell membranes which ultimately contributes to cell death^4^. Over the years, we have been using the ferroptosis pathway as therapeutic target in a phenotypic cell-based screening platform to identify potential drug candidates for the treatment of neurodegenerative diseases^5–7^. This led to the discovery of potent anti-ferroptotic compounds, two of which (J147 and CMS121) have completed Phase 1 clinical trials for Alzheimer’s Disease^8,9^.

Despite its complex nature, the study of cellular senescence has become central in aging research^2,10^. Notwithstanding the challenges posed by the heterogeneous senescent cell population and the absence of universal biomarkers, accumulating evidence from *in vivo* and *in vitro* studies underscores the pivotal role of senescence in various age-related disorders, including a plethora of neurodegenerative diseases as well as atherosclerosis, and osteoarthritis^11–13^. Consequently, senescent cells have become enticing targets for therapeutic interventions aimed at mitigating age-related diseases. While numerous compounds exhibit senomorphic or senolytic properties, their clinical translation requires meticulous consideration of potential off-target toxicity, especially in geropharmacology^14,15^. Nonetheless, the promise of these interventions lies not only in their potential to prevent disease onset but also in their capacity to alleviate symptoms and promote healthy aging. Cellular senescence is characterized by irreversible cell cycle arrest triggered by prolonged stressors such as DNA damage, chemotherapy, or oxidative stress, and is accompanied by profound metabolic alterations, particularly in lipid metabolism^10,16^. Another hallmark of senescent cells is the secretion of a complex array of inflammatory signaling molecules known as the senescence-associated secretory phenotype (SASP)^17^. The components of the SASP, which includes proteins such as interleukins 1 (IL-1), 6 (IL-6), and 8 (IL-8), not only reshape local tissue microenvironments but also exert broader paracrine effects on organ function and systemic health. Moreover, this paracrine process can propagate the senescent phenotype to otherwise healthy cells, amplifying tissue dysfunction and contributing to age-related physiopathology^18,19^.

Interestingly, senescent cells display significant alterations in lipid metabolism compared to normal cells, including accumulation of lipid droplets, increased lipid uptake, altered fatty acid (FA) composition and changes in lipid biosynthesis^20^. In addition, they present an altered membrane composition, with shifts in phospholipid profiles, increased saturation of FAs, and reduced membrane fluidity, which impair membrane signaling and contribute to their pro-inflammatory state^16,21,22^. Several enzymes associated with lipid metabolism have been found to be over-expressed in senescent cells, including acyl-CoA synthetase long-chain family member 4 (ACSL4) and acid ceramidase (ACase, ASAH1)^23,24^. While ACSL4 has been implicated in ferroptosis^25,26^, the role of ACase in this type of cell death has never been explored. Using the human fetal lung Wi-38 fibroblasts model of senescence induced by replicative exhaustion, the present study shows a previously uncharacterized role of ACase in modulating ferroptosis through alterations in cellular lipid composition. We demonstrate that ACase upregulation with senescence drives an increased sensitivity to ferroptosis and that inhibition of ACase expression significantly attenuates ferroptosis independently of the GPx4/GSH axis and iron regulation by decreasing the levels of PUFAs crucial for ferroptosis in membrane phospholipids, unveiling a key role for this enzyme in sensitizing senescent cells to this form of regulated cell death. Furthermore, our findings reveal a paracrine cell non-autonomous induction of ACase expression and ferroptosis sensitization in proliferative cells by the elements of the SASP, uncovering a previously unexplored relationship between senescent and proliferative cells in the context of ferroptosis with implications for aging tissue health. Together, these findings provide a further understanding of the relationship between ferroptosis and cellular senescence, potentially highlighting novel therapeutic targets for the modulation or removal of senescent cells.

## Results

### Lipid peroxidation and ferroptosis sensitivity increase as cells become senescent with replicative exhaustion

**Figure 1.**
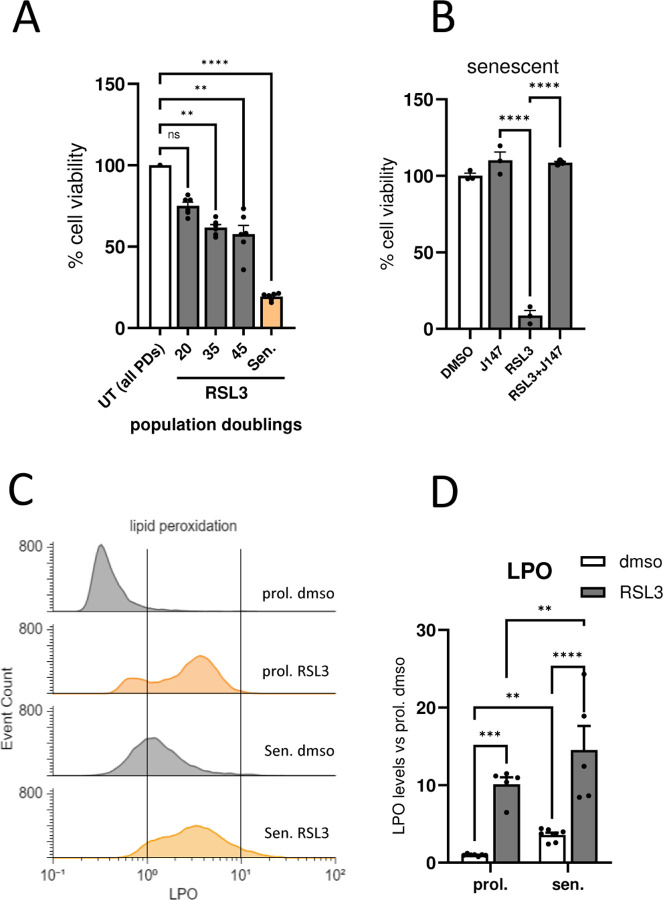
Senescent cells are more sensitive to ferroptosis. (A) Percentage of survival of cell populations of increasing PDL and senescent (sen.) cells after RSL3 (250nM) treatment. (B) Percentage of cell survival of senescent cells after RSL3 (250nM) and/or J147 (1μM) treatment. (**p < *0.01*, ****p < *0.0001*. One-way ANOVA) Representative flow cytometry histograms (C) and quantification (D) showing LPO levels (C11-Bodipy 581/591) in proliferative (prol.) and senescent (sen.) cells in the presence or absence of RSL3 (250nM). (**p < *0.01*, ***p < *0.001*, ****p < *0.0001*. Two-way ANOVA). Values represent the mean ± SEM of at least 3 independent experiments. LPO, lipid peroxidation.

Several studies have suggested a dysregulation of ferroptosis in different types of senescent cells^1,27^. To evaluate the sensitivity of Wi38 cells to ferroptotic stress, we treated senescent and proliferative cells of increasing population doubling levels (PD20, PD30 and PD40) overnight with RSL3, an inhibitor of GPX4 and a well-known ferroptosis inducer. Survival against RSL3 treatment decreased significantly as the number of doublings increased, with the lowest level of survival observed after the cells reached senescence (Fig. 1A.). In order to confirm that the cells were dying by ferroptosis, we tested the effect of J147, a compound developed in our lab based on its ability to specifically prevent ferroptosis and lipid peroxidation (LPO)^3,28^. The effect of RSL3 was prevented by co-treating the cells with J147 (Fig. 1B), confirming that the senescent cells are undergoing ferroptosis.

Since LPO is one of the main hallmarks of ferroptosis, its levels were quantified by flow cytometry using C11-bodipy 581/591 as a probe. Consistent with the observed greater sensitivity to ferroptotic stress, senescent Wi-38 cells displayed higher levels of LPO as compared to replicative cells both under control conditions and when treated with RSL3 (Fig. 1C & D). Thus, there is a clear increase in LPO and ferroptosis sensitivity with the number of cellular divisions, showing maximal levels after the cells reach replicative exhaustion and senescence.

### Acid ceramidase inhibition protects senescent cells against ferroptosis

**Figure 2.**
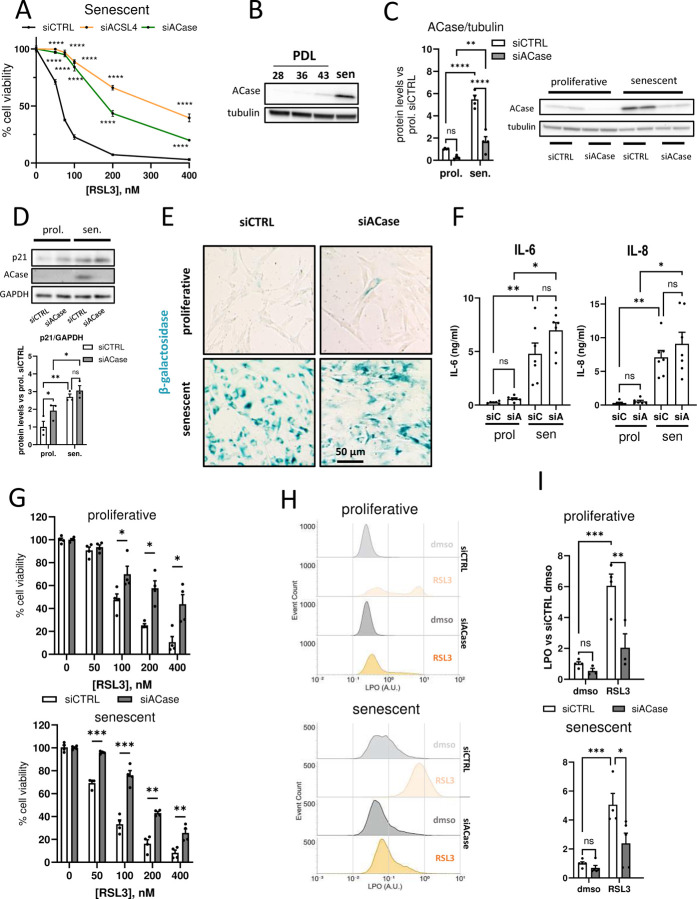
Acid ceramidase knock-down protects proliferative and senescent cells against ferroptosis independently of GSH. (A) Percentage of cell survival of senescent cells after RSL3 treatment in the presence or absence of siACSL4 or siACase (B) Representative blot of acid ceramidase protein expression in proliferative cells of increasing PDL and senescent cells. (C) Representative blot showing acid ceramidase knock-down after siRNA transfection (siACase). (D) Representative blot of p21 protein expression in the presence or absence of siACase. (E) Representative micrographs showing β-galactosidase activity (blue staining). (F) Quantification of secreted interleukin-6 and interleukin-8 levels measured by ELISA. (G) Percentage of cell survival of proliferative and senescent cells after RSL3 treatment in the presence or absence siACase. (H) Representative flow cytometry histograms and quantification (I) showing LPO levels (C11-Bodipy 581/591) in proliferative and senescent cells in the presence or absence of RSL3 (250nM) and siACase. (*p < *0.05*, **p < *0.01*, ***p < *0.001*, ****p < *0.0001*. Two-way ANOVA). Values represent the mean ± SEM of at least 3 independent experiments. PDL, population doubling level; siC, control siRNA; siA, ACase siRNA; LPO, lipid peroxidation.

Fatty acids (FAs) are the primary substrates of LPO^29^. Some studies have reported the overexpression of the FA-related enzymes ACSL4 and ACase in senescent cells^23,24^. This prompted us to ask whether these enzymes are involved in the increased sensitivity to ferroptosis observed in senescent cells. In fact, the knockdown (KD) of either ACSL4 or ACase with specific siRNAs (siACSL4; siACase) strongly protected senescent cells against RSL3 compared to a control siRNA (siCTRL) (Fig. 2A). While the role of ACSL4 in ferroptosis is well stablished and has been extensively described in the literature^25,26,30^, the involvement of ACase in this type of cell death was, to our knowledge, undocumented. For this reason, we chose to concentrate our investigation on the contribution of ACase to ferroptosis sensitivity in senescent cells. ACase breaks down ceramide into sphingosine and fatty acids, playing a key role in lipid metabolism and affecting the abundance of specific lipidic species known to be relevant to ferroptosis (i.e. sphingomyelin, ceramide-1-phosphate or fatty acids)^31–33^. A strong overexpression of ACase in senescent Wi-38 cells compared to replicative cells (5- to 20-fold) was confirmed by western blot (Fig. 2B). After 72 hours of siACase transfection, the levels of ACase were undetectable in replicative cells and strongly, but not completely, reduced in senescent cells, consistent with previous reports showing an increased stability of ACase in senescent cells (Fig. 2C)^23^. Senescent cells transfected with siACase still displayed senescence-like phenotypes, namely, high levels of p21 expression (Fig. 2D), high levels of β-galactosidase activity (Fig. 2E) and significantly increased secretion of IL-6 and IL-8 (Fig. 2F), two of the best-known cytokines contributing to the SASP^34,35^, compared to cells transfected with siCTRL. Notably, both senescent and proliferative Wi-38 cells transfected with siACase were significantly more resistant to ferroptosis induced by RSL3 as compared to cells transfected with siCTRL (Fig. 2G). The protection observed after the knockdown of ACase was additionally confirmed by treating proliferative and senescent cells with ARN14794, a specific ACase chemical inhibitor^36^. ARN14794 protected against RSL3 as well as erastin which induces ferroptosis upstream of GPX4 inhibition (Fig S1A & B). In addition, ARN14794 treatment also prevented RSL3- and erastin-induced ferroptosis in neuronal-derived murine HT22 cells, a well-studied cell model of ferroptosis (Fig S1C).

We then asked whether the protection by ACase KD correlated with a decrease in LPO. C11-Bodipy 581/591 was used to quantify the levels of LPO in replicative and senescent cells transfected with siCTRL or siACase. As measured by flow cytometry, RSL3-induced LPO was significantly reduced in siACase cells compared with siCTRL cells (Fig. 2H & I) in both proliferative and senescent cells, correlating with an increased resistance to ferroptosis.

Taken together, these data indicate a clear role of ACase in sensitizing cells to ferroptosis, especially senescent cells, where the protein is strongly overexpressed.

### The protection exerted by ACase inhibition is independent of both GPX4/GSH axis and iron

**Figure 3.**
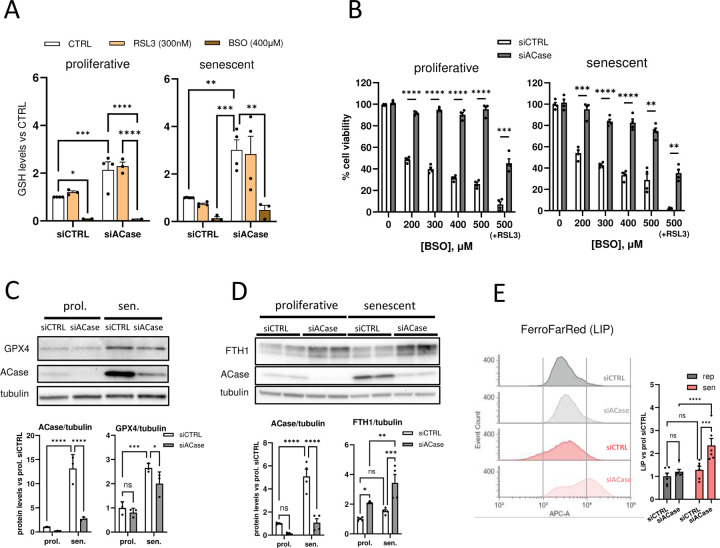
Acid ceramidase knock-down decreases lipid peroxidation independently of Fe^2+^ regulation. (A) Quantification of GSH levels in proliferative and senescent cells after the indicated treatments. (B) Percentage of cell survival of proliferative and senescent cells after GSH depletion by BSO treatment in the presence or absence siACase. Co-treatment with RSL3 is indicated (200nM). (C) Representative blot and quantification (bar graphs) of GPX4 protein expression in proliferative and senescent cells in the presence or absence of siACase. (D) Representative blot and quantification (bar graphs) of FTH1 protein expression in proliferative and senescent cells in the presence or absence of siACase. (E) Representative flow cytometry histograms and quantification (bars graph) showing labile iron pool levels (FerroFarRed) in proliferative and senescent cells in the presence or absence of siACase. (*p < *0.05*, **p < *0.01*, ***p < *0.001*, ****p < *0.0001*. Two-way ANOVA). Values represent the mean ± SEM of at least 3 independent experiments. GSH, glutathione; LIP, labile iron pool.

To explore the anti-ferroptotic mechanism underlying the effects of ACase KD, we first determined the levels of GSH in these cells. When GSH is depleted, GPX4 cannot function effectively and a cascade leading to increased LPO and ferroptosis is initiated^4^. Notably, the silencing of ACase expression, albeit incomplete in senescent cells, induced a significant increase in total GSH levels in both proliferative and senescent cells (Fig. 3A). This observation prompted us to ask whether the increase in GSH was involved in the inhibition of ferroptosis by ACase KD. To address this question, cells were treated with buthionine sulfoximine (BSO), a common ferroptosis inducer that inhibits gamma-glutamylcysteine synthetase, the rate limiting enzyme in GSH synthesis^4^. BSO depleted GSH levels in all conditions (Fig. 3A), causing significant cell death in cells transfected with siCTRL (Fig. 3B). Strikingly, ACase KD conferred strong resistance against GSH depletion, protecting cells even after RSL3 treatment (Fig. 3B). In line with the higher levels of GSH observed in senescent cells, GPX4 protein levels were also significantly increased in these cells compared to proliferative cells (Fig. 3C), possibly as part of the response to a higher level of ferroptotic stress. However, despite conferring a strong protection against ferroptosis, ACase KD significantly reduced GPX4 protein expression in senescent cells (Fig. 3C). These observations suggest that the protection by ACase KD is independent of the GPX4/GSH axis and that both the increase in GSH levels and the decrease is GPX4 expression could be the consequence of an attenuation of ferroptotic pressure.

Another factor that plays a critical role in ferroptosis is ferrous iron (Fe^2+^) which, through the Fenton reaction, generates hydroxyl radicals that initiate LPO and also acts as a co-factor to catalyze the enzymatic peroxidation of PUFAs by lipoxygenases (LOX)^4,29^. The labile iron pool (LIP), which consists of loosely bound, chemically reactive Fe^2+^, is considered the primary source of iron for ferroptosis^29^. To reduce its toxicity, the ferritin protein complex buffers the excess Fe^2+^ by oxidizing and storing it into a less reactive ferric form (Fe^3+^)^37^. To explore a possible role of ferritin in ACase protection, the levels of ferritin heavy chain 1 (FTH1) were analyzed by western blot. Notably, as seen in Fig. 3D, FTH1 levels were significantly increased after ACase KD in both proliferative and senescent cells. To test whether the observed increase in FTH1 was causing a drop in the LIP levels that could explain, at least in part, the anti-ferroptotic effect of ACase KD, the LIP levels were measured by flow cytometry using the FerroFarRed^™^ dye that stains Fe^2+^ but not Fe^3+^, and reflects the intracellular LIP levels^38–40^. Surprisingly, although they remained unchanged in proliferative cells, LIP levels were significantly increased in senescent cells after ACase KD, despite its anti-ferroptotic effect (Fig. 3E). Similar to the effects on the GPX4/GSH axis, the increased LIP levels detected after ACase KD occur in the context of an attenuation of ferroptotic stress suggesting that under these conditions Fe^2+^ might be underutilized, thereby increasing its levels. Furthermore, this increase in LIP levels likely leads to the observed upregulation of FTH1. The much stronger increase in Fe^2+^ levels in senescent cells after ACase inhibition could allow for the observable increase in LIP levels in these cells but not in proliferative cells (Fig. 3E), where the upregulation of ferritin might be sufficient to buffer the more moderate increase in Fe^2+^.

Together, these results suggest that the inhibition of ACase confers protection against ferroptosis independently of both the GPX4/GSH axis and the regulation of the intracellular Fe^2+^ levels, and that the mechanisms underlying the protection are likely upstream of these two critical elements of the ferroptosis pathway.

### ACase upregulation favors a pro-ferroptotic lipid profile in senescent cells, which is reverted after ACase inhibition

**Figure 4.**
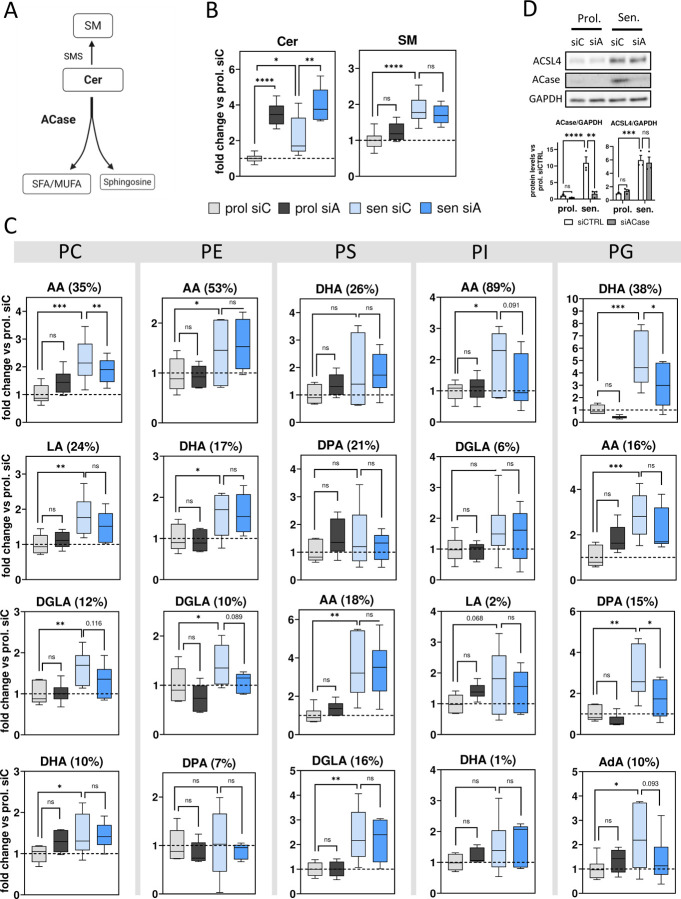
Senescent cells show a general increase in PUFA-containing phospholipids compared to proliferative cells and this is countered by acid ceramidase inhibition. (A) Diagram representing the enzymatic relation between Cer, SM, sphingosine and FAs. (B) Relative fold change of ceramide and sphingomyelin in the indicated conditions. (C) Relative fold change of the most abundant (≥ 80% of total) ^sn-2^PUFA organized by PL class and ordered by abundance inside each PL class (%) from top to bottom. (*p < *0.05*, **p < *0.01*, ***p < *0.001*, ****p < *0.0001*. One-way ANOVA). (D) Representative blot and quantification (bar graphs) of ACSL4 protein expression., (**p < *0.01*, ***p < *0.001*, ****p < *0.0001*. Two-way ANOVA). Values represent the mean ± SEM of at least 3 independent experiments. Cer, ceramide; SM, sphingomyelin; SFA/MUFA, saturated fatty acids/monounsaturated fatty acids; CerS, ceramide synthase; SMS, sphingomyelin synthase; prol, proliferative; sen, senescent; siC, CTRL siRNA; siA, ACase siRNA; AA, arachidonic acid; DHA, docosahexaenoic acid; DPA, docosapentanoic acid; DGLA, dihomo-gamma-linoleic acid; LA, linoleic acid; PC, phosphatidylcholine; PE, phosphatidylethanolamine; PS, phosphatidylserine; PI, phosphatidylinositol; PG, phosphatidylglycerol.

The roles of the GPX4/GSH axis or iron in the execution of ferroptosis greatly depend on the dynamic regulation in the levels of membrane phospholipids (PLs), specifically those containing PUFAs (PL-PUFAs), which play a crucial role in this pathway as the substrates of LPO^41^. Comprehensive profiling of various lipidic species relevant to ferroptosis, including phosphatidylcholines (PCs), phosphatidylethanolamines (PEs), phosphatidylserines (PSs), phosphatidylglycerols (PGs), phosphatidylinositols (PI), ceramides (Cer) and sphingomyelins (SMs), showed significant differences in both the abundance and composition of these lipids between proliferative and senescent cells both before and after ACase KD (Fig. 4).

Cer synthesis directly consumes free saturated (SFA) and monounsaturated FAs (MUFA), diverting them away from PL-PUFA production pathways^42–45^. Conversely, Cer breakdown by ACase releases FAs that can be used in lipid synthesis pathways, including those for PUFAs and PL-PUFAs^42,46^ (Fig. 4A). Interestingly, despite overexpressing ACase, senescent cells presented higher amounts of Cer species compared to proliferative cells (Fig. 4B & Fig S2A). As expected, siACase induced an increase in Cer compared to control siRNA in both senescent and proliferative cells (Fig. 4B & Fig S2A). Sphingomyelin (SM) species were also present in higher levels in senescent cells as compared to proliferative cells (Fig. 4B & Fig S2A). Although most SMs are synthesized by various SM synthases using Cer as a substrate^47,48^, siACase transfection did not substantially change SM levels in senescent cells, although a slight increasing trend in proliferative cells was detected. This observation could indicate a possible saturation of SM synthase in senescent cells^49^ (Fig. 4A).

PUFAs in the sn-2 position (^sn-2^PUFA) of a PL are highly susceptible to LPO and are the main substrate for LPO during ferroptosis^50^. These include Omega-6 FAs like linoleic acid (LA, 18 carbons and 2 double bonds, 18:2), dihomo-gamma-linolenic acid (DGLA, 20:3), arachidonic acid (AA, 20:4) or adrenic acid (AdA, 22:4), and Omega-3 FAs like eicosapentaenoic acid (EPA, 20:5), docosapentanoic acid (DPA, 22:5) or docosahexaenoic acid (DHA, 22:6). Although a high day-to-day variability was observed, the lipidomic analysis showed a general increasing trend in the most abundant ^sn-2^PUFA-containing PLs in senescent cells compared to proliferative cells (Fig. 4C & Fig. S2), with significant increases in all AA-containing PLs, as well as PC-LA, PC-DGLA, PC-DHA, PE-DHA, PE-DGLA, PS-DGLA, PG-DHA and PG-AdA. Importantly, ACase-KD senescent cells displayed a general decrease across the most abundant PL-PUFA species compared to control siRNA senescent cells (Fig. 4C & Fig. S2). This effect was statistically significant for PC-AA, PG-DHA and PG-DPA and showed a strong trend for PC-DGLA (*p=0.116*), PE-DGLA (*p=0.089*), PI-AA (*p=0.091*) and PG-AdA (*p=0.093*) (Fig. 4C & Fig. S2). Changes in PL-PUFAs were less consistent in proliferative cells after siACase transfection, possibly due to the lower basal levels combined with a high day-to-day variability. Similarly, a significant increase in PL containing SFA/MUFA was observed in senescent cells compared to proliferative cells, especially PC-, PS- and PG-SFA/MUFA (Fig. S2G). Additionally, albeit statistically non-significant, siACase senescent cells displayed a decreasing trend in the levels of PL-SFA/MUFA compared to siCTRL cells.

ACSL4 is a well-known driver of PUFA esterification and incorporation into PL-PUFAs in cell membranes and it plays a critical role in the execution of ferroptosis^25^. Therefore, we asked whether the decrease in PL-PUFAs observed in ACase KD senescent cells could be the consequence of a possible indirect inhibition of ACSL4 expression. As shown in Figure 4D, siACase transfection did not alter ACSL4 protein expression in either proliferative or senescent cells, suggesting that the observed reduction in PL-PUFA levels following ACase KD occurs independently of ACSL4.

Taken together, these data demonstrate that knockdown of ACase increases the Cer pool, and thereby the number of FAs incorporated into Cer, which could decrease the overall number of FAs available for the synthesis of PUFAs and their incorporation into membrane PLs. The decrease in the most abundant PL-PUFAs observed after ACase KD supports the hypothesis that ACase upregulation in senescent cells favors a pro-ferroptotic lipid profile compared to proliferative cells and could explain the increased sensitivity to ferroptosis of senescent cells and the protection by ACase inhibition by decreasing the levels of LPO substrates in cell membranes.

### Cell non-autonomous cytokine signaling from senescent cells increases ACase expression and ferroptosis sensitivity in proliferative cells

**Figure 5.**
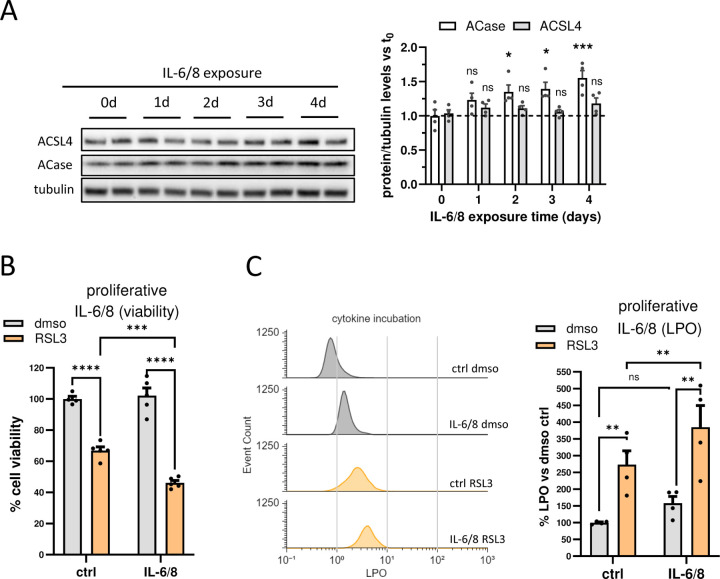
Paracrine signaling from senescent cells increases ACase expression and ferroptosis sensitivity in proliferative cells. (A) Representative blot and quantification (bar graphs) of ACase and ACSL4 protein expression of proliferative cells incubated with IL-6/8 (50/25 ng/ml) for the indicated times. (*p < *0.05*, ***p < *0.001*. Two-way ANOVA). (B) Percentage of cell survival of proliferative cells after RSL3 treatment (250nM) in the presence or absence of IL-6/8 (72 h). (C) Representative flow cytometry histograms and quantification (bar graphs) showing LPO levels (C11-Bodipy 581/591) in proliferative cells after RSL3 treatment (250nM) in the presence or absence of IL-6/8, (**p < *0.01*, ***p < *0.001*, ****p < *0.0001*. Two-way ANOVA). Values represent the mean ± SEM of at least 3 independent experiments. sen, senescent; LPO, lipid peroxidation.

It is well known that senescent cells can affect healthy neighbor cells within the reach of the paracrine effect of the SASP, inducing certain senescent-like phenotypes in proliferative cells^51^. To explore whether the changes in the regulation of ACase and ferroptosis sensitivity that we found in senescent cells could indeed spread to non-senescent cells, proliferative cells were incubated for 72 hours with a combination of recombinant human interleukin-6 and interleukin-8 (IL-6/8), two of the most representative SASP cytokines that are often used synergistically to mimic the paracrine effect of the SASP and other inflammatory signals^52–54^. Interestingly, while this treatment did not affect the expression levels of ACSL4, IL-6/8 did induce a significant time-dependent increase in ACase expression (Fig. 5A). We next asked whether cytokines from the SASP could increase the sensitivity of proliferative cells to RSL3-induced LPO and ferroptotic cell death. As shown in Fig. 5B, proliferative cells incubated with IL-6/8 were significantly more sensitive to RSL3-induced cell death than control cells. As expected, this increase in cell death correlated with elevated levels of LPO, as measured by flow cytometry (Fig. 5C). The paracrine effect of the SASP was confirmed by using a co-culture cell model with well inserts whereby proliferative cells shared media with senescent cells without direct physical contact, allowing for the subsequent separation and assessment of the proliferative population (Fig. S3A). Proliferative cells co-cultured with inserted senescent cells for 72 hours presented significantly higher levels of LPO and were more sensitive to RSL3-induced cell death than cells co-cultured with control proliferative cells (Fig. S3B & C), recapitulating the effects observed after IL-6/8 incubation.

Overall, these data suggest that the paracrine effect of cytokines such as IL-6/8 secreted by senescent cells induces senescent-like features in proliferative cells that sensitize them to ferroptosis including increased ACase protein levels and exacerbation of LPO production.

## Discussion

**Figure 6.**
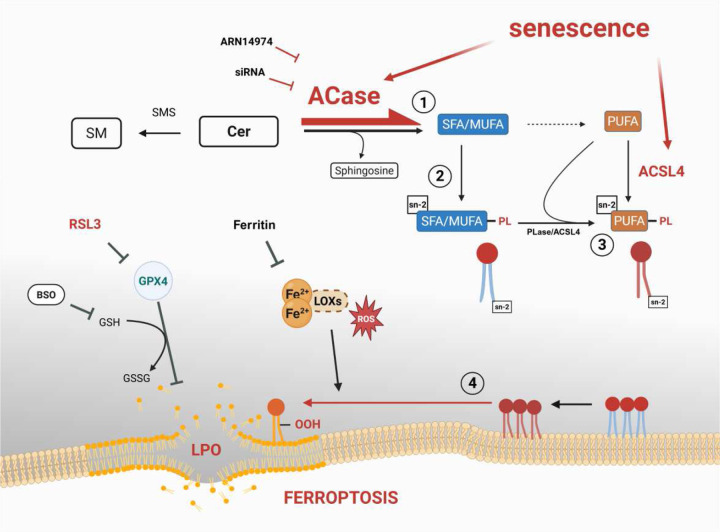
Working model illustrating the possible interplay between ACase and ferroptosis in senescent cells. The upregulation of ACase in senescent cells would increase the pool of SFA/MUFA from the Cer breakdown (1). Through the Kennedy pathway (2) and/or the Lands cycle (3), increased ^sn-2^SFA/PUFA-PL would lead to an accumulation of ^sn-2^PUFA-PL, priming the cell membranes for LPO and ferroptosis (4). SM, sphingomyelin; Cer, ceramide; SFA, saturated fatty acid; MUFA, monounsaturated fatty acid; PUFA, polyunsaturated fatty acid; PL, phospholipid; ROS, reactive oxygen species; LPO, lipid peroxidation; GSH, reduced glutathione; GSSG, oxidized glutathione.

Mixed reports on ferroptosis in senescent cells can be found in the literature. Several studies have reported higher resistance of some senescent cells to ferroptotic cell death due to disrupted ferritin and iron regulation^27,55,56^. On the other hand, recent reports show a pro-ferroptotic signaling activated upon senescence in vascular smooth muscle and an increased sensitivity to ferroptosis of senescent kidney tubular cells^57,58^. Consistent with the latter reports, our results clearly indicate that senescent human embryonic Wi-38 lung fibroblasts have elevated LPO and higher sensitivity to ferroptosis upon RSL3 treatment, compared to proliferative cells. Senescent cells can display elevated PUFA content and dysregulated lipid homeostasis as a consequence of their metabolic reorganization, which may drive enhanced susceptibility to ferroptosis^51,59^. Here we show that ACase upregulation in senescent cells plays a significant role in creating a pro-ferroptotic lipid profile that can explain the observed higher sensitivity to ferroptosis (Fig. 6).

Our results reveal that the protection exerted by ACase KD is independent of GSH. Similarly, the modulation of LIP levels does not play a role in this protection, since labile Fe^2+^ is significantly elevated after ACase inhibition, which is typically a pro-ferroptotic feature. Interestingly, the reports showing a higher resistance of senescent cells to ferroptosis also report a strong accumulation of iron^27,55,56^. GSH participates as a key part of the enzymatic defense against LPO via the action of GSH-peroxidases such as GPX4, which use GSH to reduce lipid hydroperoxides^29^. In this context, GSH is depleted as it is consumed, hence decreases in LPO are often a consequence of higher GSH availability. However, although we observed a steep increase in GSH levels after the inhibition of ACase, our data show that siACase protects equally well against ferroptosis in GSH-depleted cells. This is consistent with the idea that ACase KD induces an anti-ferroptotic state where there is less demand for GSH-dependent antioxidant activity. Such a state could be driven by a decrease in the PUFA content of the cell, which would result in less substrate for LPO, decreasing the oxidative burden on GSH and GSH-dependent enzymes^60,61^. Similarly, the increased LIP levels observed in ACase KD cells, which typically translates into elevated ferroptosis sensitivity, may also indicate a reduced availability of Fe^2+^-dependent LPO substrates. A relatively rapid shift in the PL-PUFA availability could lead to a downregulation of proteins involved in LPO, such as lipoxygenases. Since these enzymes require Fe^2+^ as a co-factor, a decrease in their expression might also contribute to elevated LIP levels. Indeed, recent studies show that substrate availability can influence the expression levels of lipoxygenases in human cells^62–64^.

When synthesized *de novo*, membrane phospholipids are initially formed with SFAs or MUFAs at the sn-1 and sn-2 positions often using FAs derived mainly from the pool of free SFAs and MUFAs in the cell in a process known as Kennedy pathway^65–67^ (Fig. 6). These free FAs are activated to acyl-CoAs by SFA/MUFA-specific acyl-CoA synthetases (e.g., ACSL enzymes) before being added to PLs^68,69^. Through the action of phospholipases like PLA2 that primarily removes the ^sn-2^FAs and the activation of free PUFAs by ACSL4, the ^sn-2^SFA- and ^sn-2^MUFA-PLs are remodeled into ^sn-2^PUFA-PLs, a pathway known as the Lands cycle^30,70^. The cellular pool of free SFA/MUFAs can originate from *de novo* lipogenesis, exogenous intake or lipolysis of stored lipids such as ceramides^70^. The strong overexpression of ACase observed in senescent cells, along with an increase in total ceramides compared to proliferative cells (Fig. 4B), could indeed amplify the release of free SFA/MUFAs by the lipolysis pathway. Higher levels of SFA/MUFA-PLs could confer resistance against ferroptosis in some contexts by competing with PUFAs for membrane incorporation, making membranes more rigid and less prone to oxidation^71,72^. However, their excess has also been reported to promote PUFA-PL accumulation through Lands cycle exchange, increasing vulnerability to peroxidation and ferroptosis^71,73^. Consistent with this scenario, we observed increased levels of both SFA/MUFA-PLs and PUFA-PLs in senescent cells along with higher LPO levels (Fig. 6).

PC and PG displayed the most significant changes between proliferative and senescent cells as well as after ACase inhibition. PC is the most abundant PL in eukaryotic membranes, including the mitochondria and the outer leaflet of the plasma membrane and ^sn-2^PUFA-PCs are major contributors to ferroptosis^70^. Conversely, PG is a less abundant PL and its contribution to ferroptosis is largely unknown. However, direct strong oxidation of ^sn-2^PUFA-PGs during ferroptosis has been reported^74^. PGs are highly enriched in mitochondria, especially the inner membrane, where it is rapidly transformed into cardiolipin (CL) and supports protein translocation or respiratory chain activity^75^. PUFA-CL peroxidation has been proposed as a marker of mitochondrial LPO^76^. Given the critical role of mitochondria during ferroptosis^77,78^, the role of ^sn-2^PUFA-PGs, and possibly CL, could be of special relevance in the context of senescence and warrants further investigation.

ACSL4 plays a critical role in the regulation of PL-PUFA composition^25,62,63^. Numerous studies have demonstrated that ACSL4 is critical for the execution of ferroptosis, and it is upregulated during senescence^24,25^. High ACSL4 expression increases the PL-PUFA content, and thus the readily oxidizable lipid pool and ferroptosis sensitivity. In fact, ACSL4-KD protects senescent cells against RSL3 in our model. Despite the observed decrease in PL-PUFA content and LPO after ACase inhibition, our results showed that ACSL4 protein levels remain high in senescent cells following ACase inhibition, suggesting that the downregulation of the SFA/MUFA-PLs supply could play a role in the decrease of PUFA-PLs independently of ACSL4. In addition, the increase in ceramide content observed after ACase KD could also limit the pool of available free SFAs and MUFAs for other anabolic lipid processes, including elongation/desaturation and PUFA re-acylation by ACSL4^70^. Nonetheless, while our findings show a strong association between ACase inhibition and decreased membrane PL-PUFA levels, future studies should further explore the underlying biochemical pathways driving this effect.

The SASP can induce paracrine effects, influencing nearby cells, thereby altering the local tissue environment^17,35,79^. One prominent paracrine effect is the induction of senescence in neighboring healthy, non-senescent cells, a process known as paracrine or bystander senescence^80^. This occurs through SASP factors that trigger DNA damage responses or activate pathways that lead to senescence thereby potentially amplifying tissue dysfunction over time. Here we found that Wi-38 senescent cells can induce a significant increase in ACase expression in proliferative cells through the SASP, with IL-6 and IL-8 being sufficient to exert this response. Importantly, we observed a significant increase in the sensitivity to LPO and ferroptotic cell death of non-senescent cells after IL-6/8 incubation or co-culture with senescent cells. This finding further highlights the potential damage through propagation that can arise from the accumulation of senescent cells during disease or aging triggering not only senescence of bystander cells but also increased sensitivity to ferroptotic death.

Growing evidence indicates that a gradual increase in ferroptotic-related stress, even in the absence of cell death, can be a consequence of the aging process itself^81^. For this reason, eliminating senescent cells may in turn contribute to a general reduction in the ferroptotic-related stress of aging tissues. Indeed, several studies have explored the increased susceptibility of senescent cells to ferroptosis as a strategy for their clearance^82^. However, our data indicates a further complexity in the interplay between cell survival, senescence and cancer. For example, ACase has been shown to promote the survival of senescent cells by controlling ceramide levels and increasing sphingosine and sphingosine-1-phosphate (S1P) levels, both of which are associated with cell survival and reduced apoptosis^23^. This primary metabolic shift could be causing susceptibility to ferroptosis as an unintended secondary effect. Conversely, ACase activity has been found to contribute to tumorigenesis in melanoma cells and to confer resistance to radiotherapy in prostate cancer cells^83,84^, and ACase is overexpressed in various malignancies^85–87^. As a matter of fact, cancer cells often display a higher susceptibility to ferroptosis, due to their elevated iron content, high oxidative stress and altered lipid metabolism, and numerous studies have explored the induction of ferroptosis as an approach to treating cancer for this reason^88,89^. Therefore, further studies exploring in depth the modulation of ACase and ferroptosis in the context of aging could be highly worthwhile.

Altogether, the present study provides not only further evidence for an increased sensitivity of senescent cells to ferroptosis but also a deeper understanding of the mechanisms underlying this change. In addition to expanding our knowledge about how changes in lipid metabolism associated with senescence can influence the sensitivity to cell death, these findings open new avenues for the development of effective senolytic drugs that attenuate or counteract the deleterious effects of senescent cells during aging.

## Materials and methods

### Materials

ELISA kits for IL-6 (#88-7066-77) and IL-8 (#88-8086-77) were purchased from Life Technologies. Human IL-6 (#20006) and IL-8 (#20008) recombinant proteins were purchased from PeproTech. Ferroptosis inducers erastin (#HY-15763) and RSL3 (#HY-100218A) were purchased from MedChemExpress. Buthionine-sulfoximine (BSO) was purchased from Sigma (#B2515); ARN14794 was purchased from Cayman Chemical (#17119).

### Cell cultures and replicative senescence

Human Wi-38 embryonic lung fibroblasts (Coriell Institute) and HT22 mouse hippocampal nerve cells were cultured in high-glucose Dulbecco’s modified Eagle’s medium (DMEM) (#11995065, Invitrogen) supplemented with 10% fetal bovine serum (FCS) (#SH30073, Hyclone) and incubated at 37 °C in 10% CO_2_. Wi-38 cells with a population doubling level (PDL) of 20 were thawed and grown under standard conditions. Cells were counted and passaged when they reached 70–80% confluency. Cells stopped dividing and became senescent at PDL 55–60. Cells ranging between PDL30 and PDL40 were used as control proliferative cells. After the cells stopped dividing, senescence-associated β-galactosidase staining (#9860, Cell Signaling), p21 protein levels and analyses of culture supernatants to detect the factors of the senescence-associated secretory phenotype (SASP) IL-6 and IL-8 were performed to characterize replicative senescent cells.

### Ferroptosis *in vitro* assay

5 × 10^3^ HT22 or Wi-38 cells were plated per well in 96 well plates and incubated overnight. The medium was then exchanged with fresh medium and RSL3 (200 nM, unless otherwise indicated) was added alone or in combination with the indicated compounds at the indicated concentrations. 24 h later, the cellular viability was measured by the 3-(4, 5-dimethylthiazolyl2)-2,5-diphenyltetrazolium bromide (MTT) assay, as previously described^90^. Cell survival was also confirmed by visual inspection of the wells.

### Measurement lipid peroxidation and labile Fe^2+^ levels by flow cytometry

7.5 × 10^4^ Wi-38 cells per well were seeded in 24 well plates. After 24 h, the indicated compounds were added and after 3–6 h, the media was aspirated and 250 μl per well of BODIPY 581/591 C11 (#D3861, Invitrogen) (1 μM) or FerroFarRed (#SCT037, Goryo Chemical) (5 μM) were added in the presence of the different compounds, to detect lipid peroxidation or labile Fe^2+^, respectively. The cells were incubated for 30 min, washed and trypsinized. The fluorescence was measured at 4 °C using a FACSymphony A3 flow cytometer (BD Biosciences, USA). LPO was calculated ratiometrically by dividing the green signal by the red signal, hence the levels of LPO were directly proportional to the green fluorescence and inversely proportional to the red fluorescence. The data were normalized to the emission of control cells treated with DMSO.

### Measurement of glutathione levels

For measurement of total glutathione (tGSH), 3 × 10^5^ Wi-38 cells were plated in 60 mm dishes. After 24 h, the medium was exchanged with fresh medium, and corresponding treatments were added. After this, the cells were scraped into ice-cold PBS, and 10% sulfosalicylic acid was added at a final concentration of 3.3%. tGSH was determined by the recycling assay based on the reduction of 5,5-dithiobis (2-nitrobenzoic acid) with glutathione reductase and NADPH^91^, and normalized to protein recovered from the acid-precipitated pellet by treatment with 0.2 N NaOH at 37 °C overnight and measured by the bicinchoninic acid assay (#23225, Pierce).

### Western blotting

Cells were washed and scraped into cold PBS. The supernatant was discarded, and cells were lysed in RIPA lysis buffer (Santa Cruz Biotechnology) containing protease/phosphatase inhibitors (Bio-Rad) and incubated on ice for 10 min. Lysates were separated by SDS-polyacrylamide gel electrophoresis (SDS-PAGE) and transferred onto PVDF membranes via semi-dry transfer (Trans-Blot^®^ Turbo, Bio-Rad) and immunoblotted with the respective antibodies (ASAH1 #sc-136275, Santa Cruz; ACSL4 # sc-271800, Santa Cruz; FTH1 #4393, Cell Signaling; GPx4 #sc-166570, Santa Cruz; p21, #37543 Cell Signaling), followed by incubations with the appropriate secondary antibodies conjugated with horseradish peroxidase (Bio-Rad). Levels of the protein of interest were normalized to housekeeping proteins (tubulin #2148, Cell Signaling; GAPDH #97166, Cell Signaling).

### siRNA transfection

Cells were seeded in 100 mm dishes at a density of 2 × 10^4^ cells. 24 h post-seeding, cells were transfected with siRNA against acid ceramidase (ACase) (ASAH1, #sc-105032, Santa Cruz,) or a negative control siRNA (#1027280, Qiagen), using lipofectamine RNAiMAX (#13778075, ThermoFisher) as the transfection reagent and Opti-MEM (#31985070, Gibco) as the transfection medium. Medium was replaced 24 h post-transfection and the cells were incubated in fresh media for 72 h. Transfection efficiency was determined via western blot.

### Lipidomics

After 72 h of control or ACase siRNA transfection, cells were trypsinized, washed and centrifuged, and the pellet was flash-frozen with liquid nitrogen and stored at −80°C. Lipidomic analysis was performed by the LIPID MAPS Lipidomics Core at the University of California, San Diego, following established protocols^92^. The analysis focused on phospholipids, ceramides, sphingomyelins, and sphingoid bases. Lipids were extracted from samples using a modified Folch method with chloroform/methanol (2:1, v/v). The extracted lipids were then analyzed using a combination of shotgun and LC-based lipidomics approaches. Data processing and quantification were performed using specialized software, with multiple standards including internal standards and endogenous abundance species employed for accurate quantitation. The analysis provided comprehensive profiling and quantification of the lipid species present in the samples. Statistical and pathway analysis were performed using Python and GraphPad software.

### Cytokines and co-culture experiments

1.5 × 10^4^ proliferative cells (PDL 40–45) per well were seeded in 24 well plates. After attachment, the cells were incubated for 96 h with a combination of IL-6/IL-8 (2:1 ratio, 50/25 ng/ml), based on previous reports of a synergistic IL-6 and IL-8 paracrine effect^52–54^. For the co-culture experiments, inserts (#662641, Greiner) seeded with 4.5 × 10^4^ senescent cells or 1.5 × 10^4^ proliferative cells (control) were placed in the wells instead of IL-6/8. After the treatments, the media with cytokines or the inserts were removed and the remaining cells in the well were washed before being tested for protein analysis, cell survival or LPO quantification.

### Quantification and statistical analysis

Statistical analysis was performed using GraphPad Prism 8 and a p value of <0.05 was considered as significant. The data representation and statistical tests applied are described in the figure legends.

## Supplementary Material

1

## References

[1] Coradduzza D. Ferroptosis and Senescence: A Systematic Review. Int J Mol Sci 24, doi:10.3390/ijms24043658 (2023). PMC9963234 36835065

[2] Gorgoulis V. Cellular Senescence: Defining a Path Forward. Cell 179, 813–827, doi:10.1016/j.cell.2019.10.005 (2019). 31675495

[3] Soriano-Castell D., Currais A. & Maher P. Defining a pharmacological inhibitor fingerprint for oxytosis/ferroptosis. Free Radic Biol Med 171, 219–231, doi:10.1016/j.freeradbiomed.2021.05.023 (2021). 34010663 PMC8217321

[4] Tan S., Schubert D. & Maher P. Oxytosis: A novel form of programmed cell death. Curr Top Med Chem 1, 497–506, doi:10.2174/1568026013394741 (2001). 11895126

[5] Currais A. Modulation of p25 and inflammatory pathways by fisetin maintains cognitive function in Alzheimer’s disease transgenic mice. Aging Cell 13, 379–390, doi:10.1111/acel.12185 (2014). 24341874 PMC3954948

[6] Maher P. ERK activation by the polyphenols fisetin and resveratrol provides neuroprotection in multiple models of Huntington’s disease. Hum Mol Genet 20, 261–270, doi:10.1093/hmg/ddq460 (2011). 20952447 PMC3005900

[7] Maher P., Salgado K. F., Zivin J. A. & Lapchak P. A. A novel approach to screening for new neuroprotective compounds for the treatment of stroke. Brain Res 1173, 117–125, doi:10.1016/j.brainres.2007.07.061 (2007). 17765210 PMC2111291

[8] Prior M., Dargusch R., Ehren J. L., Chiruta C. & Schubert D. The neurotrophic compound J147 reverses cognitive impairment in aged Alzheimer’s disease mice. Alzheimers Res Ther 5, 25, doi:10.1186/alzrt179 (2013). 23673233 PMC3706879

[9] Ates G., Goldberg J., Currais A. & Maher P. CMS121, a fatty acid synthase inhibitor, protects against excess lipid peroxidation and inflammation and alleviates cognitive loss in a transgenic mouse model of Alzheimer’s disease. Redox Biol 36, 101648, doi:10.1016/j.redox.2020.101648 (2020). 32863221 PMC7394765

[10] Childs B. G. Senescent cells: an emerging target for diseases of ageing. Nat Rev Drug Discov 16, 718–735, doi:10.1038/nrd.2017.116 (2017). 28729727 PMC5942225

[11] Bussian T. J. Clearance of senescent glial cells prevents tau-dependent pathology and cognitive decline. Nature 562, 578–582, doi:10.1038/s41586-018-0543-y (2018). 30232451 PMC6206507

[12] Childs B. G. Senescent intimal foam cells are deleterious at all stages of atherosclerosis. Science 354, 472–477, doi:10.1126/science.aaf6659 (2016). 27789842 PMC5112585

[13] Jeon O. H. Local clearance of senescent cells attenuates the development of post-traumatic osteoarthritis and creates a pro-regenerative environment. Nat Med 23, 775–781, doi:10.1038/nm.4324 (2017). 28436958 PMC5785239

[14] Chaib S., Tchkonia T. & Kirkland J. L. Cellular senescence and senolytics: the path to the clinic. Nat Med 28, 1556–1568, doi:10.1038/s41591-022-01923-y (2022). 35953721 PMC9599677

[15] Lagoumtzi S. M. & Chondrogianni N. Senolytics and senomorphics: Natural and synthetic therapeutics in the treatment of aging and chronic diseases. Free Radic Biol Med 171, 169–190, doi:10.1016/j.freeradbiomed.2021.05.003 (2021). 33989756

[16] Ogrodnik M. Cellular senescence drives age-dependent hepatic steatosis. Nat Commun 8, 15691, doi:10.1038/ncomms15691 (2017). 28608850 PMC5474745

[17] Coppe J. P. Senescence-associated secretory phenotypes reveal cell-nonautonomous functions of oncogenic RAS and the p53 tumor suppressor. PLoS Biol 6, 2853–2868, doi:10.1371/journal.pbio.0060301 (2008). 19053174 PMC2592359

[18] Gross P. S. Senescent-like microglia limit remyelination through the senescence associated secretory phenotype. Nat Commun 16, 2283, doi:10.1038/s41467-025-57632-w (2025). 40055369 PMC11889183

[19] Wang B., Han J., Elisseeff J. H. & Demaria M. The senescence-associated secretory phenotype and its physiological and pathological implications. Nat Rev Mol Cell Biol 25, 958–978, doi:10.1038/s41580-024-00727-x (2024). 38654098

[20] Millner A. & Atilla-Gokcumen G. E. Lipid Players of Cellular Senescence. Metabolites 10, doi:10.3390/metabo10090339 (2020). PMC7570155 32839400

[21] Flor A. C., Wolfgeher D., Wu D. & Kron S. J. A signature of enhanced lipid metabolism, lipid peroxidation and aldehyde stress in therapy-induced senescence. Cell Death Discov 3, 17075, doi:10.1038/cddiscovery.2017.75 (2017). 29090099 PMC5661608

[22] Quijano C. Oncogene-induced senescence results in marked metabolic and bioenergetic alterations. Cell Cycle 11, 1383–1392, doi:10.4161/cc.19800 (2012). 22421146 PMC3350879

[23] Munk R. Acid ceramidase promotes senescent cell survival. Aging (Albany NY) 13, 15750–15769, doi:10.18632/aging.203170 (2021). 34102611 PMC8266329

[24] Yao Y. Ferroptosis promotes sepsis-related lung injury via endothelial cell senescence. Cell Signal, 112018, doi:10.1016/j.cellsig.2025.112018 (2025). 40701314

[25] Doll S. ACSL4 dictates ferroptosis sensitivity by shaping cellular lipid composition. Nat Chem Biol 13, 91–98, doi:10.1038/nchembio.2239 (2017). 27842070 PMC5610546

[26] Ye Y. The ERK-cPLA2-ACSL4 axis mediating M2 macrophages ferroptosis impedes mucosal healing in ulcerative colitis. Free Radic Biol Med 214, 219–235, doi:10.1016/j.freeradbiomed.2024.02.016 (2024). 38367927

[27] Wei X. Defective NCOA4-dependent ferroptosis in senescent fibroblasts retards diabetic wound healing. Cell Death Discov 9, 138, doi:10.1038/s41420-023-01437-7 (2023). 37117222 PMC10147701

[28] Chen Q. A novel neurotrophic drug for cognitive enhancement and Alzheimer’s disease. PLoS One 6, e27865, doi:10.1371/journal.pone.0027865 (2011). 22194796 PMC3237323

[29] Dixon S. J. Ferroptosis: an iron-dependent form of nonapoptotic cell death. Cell 149, 1060–1072, doi:10.1016/j.cell.2012.03.042 (2012). 22632970 PMC3367386

[30] Qiu Y. ACSL4-Mediated Membrane Phospholipid Remodeling Induces Integrin beta1 Activation to Facilitate Triple-Negative Breast Cancer Metastasis. Cancer Res 84, 1856–1871, doi:10.1158/0008-5472.CAN-23-2491 (2024). 38471082 PMC11148537

[31] Frohbergh M., He X. & Schuchman E. H. The molecular medicine of acid ceramidase. Biol Chem 396, 759–765, doi:10.1515/hsz-2014-0290 (2015). 25938220

[32] Thayyullathil F. Acid sphingomyelinase-dependent autophagic degradation of GPX4 is critical for the execution of ferroptosis. Cell Death Dis 12, 26, doi:10.1038/s41419-020-03297-w (2021). 33414455 PMC7791123

[33] Vu N. T., Kim M., Stephenson D. J., MacKnight H. P. & Chalfant C. E. Ceramide Kinase Inhibition Drives Ferroptosis and Sensitivity to Cisplatin in Mutant KRAS Lung Cancer by Dysregulating VDAC-Mediated Mitochondria Function. Mol Cancer Res 20, 1429–1442, doi:10.1158/1541-7786.MCR-22-0085 (2022). 35560154 PMC9444881

[34] Basisty N. A proteomic atlas of senescence-associated secretomes for aging biomarker development. PLoS Biol 18, e3000599, doi:10.1371/journal.pbio.3000599 (2020). 31945054 PMC6964821

[35] Coppe J. P., Desprez P. Y., Krtolica A. & Campisi J. The senescence-associated secretory phenotype: the dark side of tumor suppression. Annu Rev Pathol 5, 99–118, doi:10.1146/annurev-pathol-121808-102144 (2010). 20078217 PMC4166495

[36] Pizzirani D. Benzoxazolone carboxamides: potent and systemically active inhibitors of intracellular acid ceramidase. Angew Chem Int Ed Engl 54, 485–489, doi:10.1002/anie.201409042 (2015). 25395373 PMC4502975

[37] Kotla N. K., Dutta P., Parimi S. & Das N. K. The Role of Ferritin in Health and Disease: Recent Advances and Understandings. Metabolites 12, doi:10.3390/metabo12070609 (2022). PMC9320524 35888733

[38] Grignano E. Dihydroartemisinin-induced ferroptosis in acute myeloid leukemia: links to iron metabolism and metallothionein. Cell Death Discov 9, 97, doi:10.1038/s41420-023-01371-8 (2023). 36928207 PMC10020442

[39] Kittilukkana A., Carmona A., Pilapong C. & Ortega R. TauSTED super-resolution imaging of labile iron in primary hippocampal neurons. Metallomics 16, doi:10.1093/mtomcs/mfad074 (2024). 38148121

[40] Miyamoto H. D. Iron Overload via Heme Degradation in the Endoplasmic Reticulum Triggers Ferroptosis in Myocardial Ischemia-Reperfusion Injury. JACC Basic Transl Sci 7, 800–819, doi:10.1016/j.jacbts.2022.03.012 (2022). 36061338 PMC9436815

[41] Kagan V. E. Oxidized arachidonic and adrenic PEs navigate cells to ferroptosis. Nat Chem Biol 13, 81–90, doi:10.1038/nchembio.2238 (2017). 27842066 PMC5506843

[42] Hannun Y. A. & Obeid L. M. Principles of bioactive lipid signalling: lessons from sphingolipids. Nat Rev Mol Cell Biol 9, 139–150, doi:10.1038/nrm2329 (2008). 18216770

[43] Kihara A. Synthesis and degradation pathways, functions, and pathology of ceramides and epidermal acylceramides. Prog Lipid Res 63, 50–69, doi:10.1016/j.plipres.2016.04.001 (2016). 27107674

[44] Luukkonen P. K. Hepatic ceramides dissociate steatosis and insulin resistance in patients with non-alcoholic fatty liver disease. J Hepatol 64, 1167–1175, doi:10.1016/j.jhep.2016.01.002 (2016). 26780287

[45] Sokol K. H. Lipid availability influences ferroptosis sensitivity in cancer cells by regulating polyunsaturated fatty acid trafficking. Cell Chem Biol 32, 408–422 e406, doi:10.1016/j.chembiol.2024.09.008 (2025). 39442523 PMC11928283

[46] Mao C. & Obeid L. M. Ceramidases: regulators of cellular responses mediated by ceramide, sphingosine, and sphingosine-1-phosphate. Biochim Biophys Acta 1781, 424–434, doi:10.1016/j.bbalip.2008.06.002 (2008). 18619555 PMC2614331

[47] Ternes P., Brouwers J. F., van den Dikkenberg J. & Holthuis J. C. Sphingomyelin synthase SMS2 displays dual activity as ceramide phosphoethanolamine synthase. J Lipid Res 50, 2270–2277, doi:10.1194/jlr.M900230-JLR200 (2009). 19454763 PMC2759833

[48] Lafont E. Caspase-mediated inhibition of sphingomyelin synthesis is involved in FasL-triggered cell death. Cell Death Differ 17, 642–654, doi:10.1038/cdd.2009.130 (2010). 19779494

[49] Taniguchi M. & Okazaki T. The role of sphingomyelin and sphingomyelin synthases in cell death, proliferation and migration-from cell and animal models to human disorders. Biochim Biophys Acta 1841, 692–703, doi:10.1016/j.bbalip.2013.12.003 (2014). 24355909

[50] Tang D., Chen X., Kang R. & Kroemer G. Ferroptosis: molecular mechanisms and health implications. Cell Res 31, 107–125, doi:10.1038/s41422-020-00441-1 (2021). 33268902 PMC8026611

[51] Wiley C. D. & Campisi J. The metabolic roots of senescence: mechanisms and opportunities for intervention. Nat Metab 3, 1290–1301, doi:10.1038/s42255-021-00483-8 (2021). 34663974 PMC8889622

[52] Barajas-Gomez B. A. Relationship of inflammatory profile of elderly patients serum and senescence-associated secretory phenotype with human breast cancer cells proliferation: Role of IL6/IL8 ratio. Cytokine 91, 13–29, doi:10.1016/j.cyto.2016.12.001 (2017). 27951455

[53] Jayatilaka H. Synergistic IL-6 and IL-8 paracrine signalling pathway infers a strategy to inhibit tumour cell migration. Nat Commun 8, 15584, doi:10.1038/ncomms15584 (2017). 28548090 PMC5458548

[54] Ortiz-Montero P., Londono-Vallejo A. & Vernot J. P. Senescence-associated IL-6 and IL-8 cytokines induce a self- and cross-reinforced senescence/inflammatory milieu strengthening tumorigenic capabilities in the MCF-7 breast cancer cell line. Cell Commun Signal 15, 17, doi:10.1186/s12964-017-0172-3 (2017). 28472950 PMC5418812

[55] Feng Y. Iron retardation in lysosomes protects senescent cells from ferroptosis. Aging (Albany NY) 16, 7683–7703, doi:10.18632/aging.205777 (2024). 38683121 PMC11131988

[56] Masaldan S. Iron accumulation in senescent cells is coupled with impaired ferritinophagy and inhibition of ferroptosis. Redox Biol 14, 100–115, doi:10.1016/j.redox.2017.08.015 (2018). 28888202 PMC5596264

[57] Liao C. M. Induction of ferroptosis selectively eliminates senescent tubular cells. Am J Transplant 22, 2158–2168, doi:10.1111/ajt.17102 (2022). 35607817

[58] Sun D. Y. Pro-ferroptotic signaling promotes arterial aging via vascular smooth muscle cell senescence. Nat Commun 15, 1429, doi:10.1038/s41467-024-45823-w (2024). 38365899 PMC10873425

[59] Das U. N. “Cell Membrane Theory of Senescence” and the Role of Bioactive Lipids in Aging, and Aging Associated Diseases and Their Therapeutic Implications. Biomolecules 11, doi:10.3390/biom11020241 (2021). PMC7914625 33567774

[60] Grim J. M., Hyndman K. A., Kriska T., Girotti A. W. & Crockett E. L. Relationship between oxidizable fatty acid content and level of antioxidant glutathione peroxidases in marine fish. J Exp Biol 214, 3751–3759, doi:10.1242/jeb.058214 (2011). 22031739 PMC3202513

[61] Christon R., Haloui R. B. & Durand G. Dietary polyunsaturated fatty acids and aging modulate glutathione-related antioxidants in rat liver. J Nutr 125, 3062–3070, doi:10.1093/jn/125.12.3062 (1995). 7500185

[62] Benatzy Y., Palmer M. A. & Brune B. Arachidonate 15-lipoxygenase type B: Regulation, function, and its role in pathophysiology. Front Pharmacol 13, 1042420, doi:10.3389/fphar.2022.1042420 (2022). 36438817 PMC9682198

[63] Snodgrass R. G. & Brune B. Regulation and Functions of 15-Lipoxygenases in Human Macrophages. Front Pharmacol 10, 719, doi:10.3389/fphar.2019.00719 (2019). 31333453 PMC6620526

[64] Horn T. Functional characterization of genetic enzyme variations in human lipoxygenases. Redox Biol 1, 566–577, doi:10.1016/j.redox.2013.11.001 (2013). 24282679 PMC3840004

[65] Wang B. & Tontonoz P. Phospholipid Remodeling in Physiology and Disease. Annu Rev Physiol 81, 165–188, doi:10.1146/annurev-physiol-020518-114444 (2019). 30379616 PMC7008953

[66] Morita S. Y. & Ikeda Y. Regulation of membrane phospholipid biosynthesis in mammalian cells. Biochem Pharmacol 206, 115296, doi:10.1016/j.bcp.2022.115296 (2022). 36241095

[67] Shindou H., Hishikawa D., Harayama T., Yuki K. & Shimizu T. Recent progress on acyl CoA: lysophospholipid acyltransferase research. J Lipid Res 50 Suppl, S46–51, doi:10.1194/jlr.R800035-JLR200 (2009). 18931347 PMC2674719

[68] Soupene E. & Kuypers F. A. Mammalian long-chain acyl-CoA synthetases. Exp Biol Med (Maywood) 233, 507–521, doi:10.3181/0710-MR-287 (2008). 18375835 PMC3377585

[69] Mashek D. G., Li L. O. & Coleman R. A. Long-chain acyl-CoA synthetases and fatty acid channeling. Future Lipidol 2, 465–476, doi:10.2217/17460875.2.4.465 (2007). 20354580 PMC2846691

[70] Kim J. W., Lee J. Y., Oh M. & Lee E. W. An integrated view of lipid metabolism in ferroptosis revisited via lipidomic analysis. Exp Mol Med 55, 1620–1631, doi:10.1038/s12276-023-01077-y (2023). 37612411 PMC10474074

[71] Liang D., Minikes A. M. & Jiang X. Ferroptosis at the intersection of lipid metabolism and cellular signaling. Mol Cell 82, 2215–2227, doi:10.1016/j.molcel.2022.03.022 (2022). 35390277 PMC9233073

[72] Magtanong L. Exogenous Monounsaturated Fatty Acids Promote a Ferroptosis-Resistant Cell State. Cell Chem Biol 26, 420–432 e429, doi:10.1016/j.chembiol.2018.11.016 (2019). 30686757 PMC6430697

[73] Bartolacci C. Targeting de novo lipogenesis and the Lands cycle induces ferroptosis in KRAS-mutant lung cancer. Nat Commun 13, 4327, doi:10.1038/s41467-022-31963-4 (2022). 35882862 PMC9325712

[74] Hassannia B. Nano-targeted induction of dual ferroptotic mechanisms eradicates high-risk neuroblastoma. J Clin Invest 128, 3341–3355, doi:10.1172/JCI99032 (2018). 29939160 PMC6063467

[75] Yang Y., Lee M. & Fairn G. D. Phospholipid subcellular localization and dynamics. J Biol Chem 293, 6230–6240, doi:10.1074/jbc.R117.000582 (2018). 29588369 PMC5925819

[76] Lyamzaev K. G., Panteleeva A. A., Simonyan R. A., Avetisyan A. V. & Chernyak B. V. The critical role of mitochondrial lipid peroxidation in ferroptosis: insights from recent studies. Biophys Rev 15, 875–885, doi:10.1007/s12551-023-01126-w (2023). 37974984 PMC10643799

[77] Gao M. Role of Mitochondria in Ferroptosis. Mol Cell 73, 354–363 e353, doi:10.1016/j.molcel.2018.10.042 (2019). 30581146 PMC6338496

[78] Tadokoro T. Mitochondria-dependent ferroptosis plays a pivotal role in doxorubicin cardiotoxicity. JCI Insight 8, doi:10.1172/jci.insight.169756 (2023). PMC10070098 36946465

[79] Krtolica A., Parrinello S., Lockett S., Desprez P. Y. & Campisi J. Senescent fibroblasts promote epithelial cell growth and tumorigenesis: a link between cancer and aging. Proc Natl Acad Sci U S A 98, 12072–12077, doi:10.1073/pnas.211053698 (2001). 11593017 PMC59769

[80] Admasu T. D. Selective ablation of primary and paracrine senescent cells by targeting iron dyshomeostasis. Cell Rep 42, 112058, doi:10.1016/j.celrep.2023.112058 (2023). 36753419

[81] Maher P., Soriano-Castell D., Dar N. J., Soriano S. & Currais A. Ferroptosis-related stress during aging and its relevance to disease. Geroscience, doi:10.1007/s11357-025-01929-7 (2025). PMC13575064 41082098

[82] Kureel S. K. & Rasmussen B. B. Targeting Ferroptosis to Eliminate Senescent Cells: Mechanisms and Therapeutic Potential. Aging Dis, doi:10.14336/AD.2025.0141 (2025). PMC13256700 40768631

[83] Lai M. Complete Acid Ceramidase ablation prevents cancer-initiating cell formation in melanoma cells. Sci Rep 7, 7411, doi:10.1038/s41598-017-07606-w (2017). 28785021 PMC5547127

[84] Mahdy A. E. Acid ceramidase upregulation in prostate cancer cells confers resistance to radiation: AC inhibition, a potential radiosensitizer. Mol Ther 17, 430–438, doi:10.1038/mt.2008.281 (2009). 19107118 PMC2835081

[85] Liu X. Acid ceramidase upregulation in prostate cancer: role in tumor development and implications for therapy. Expert Opin Ther Targets 13, 1449–1458, doi:10.1517/14728220903357512 (2009). 19874262 PMC2796572

[86] Roh J. L., Park J. Y., Kim E. H. & Jang H. J. Targeting acid ceramidase sensitises head and neck cancer to cisplatin. Eur J Cancer 52, 163–172, doi:10.1016/j.ejca.2015.10.056 (2016). 26687835

[87] Taniai T. Inhibition of acid ceramidase elicits mitochondrial dysfunction and oxidative stress in pancreatic cancer cells. Cancer Sci 112, 4570–4579, doi:10.1111/cas.15123 (2021). 34459070 PMC8586682

[88] Zhou Q. Ferroptosis in cancer: From molecular mechanisms to therapeutic strategies. Signal Transduct Target Ther 9, 55, doi:10.1038/s41392-024-01769-5 (2024). 38453898 PMC10920854

[89] Wahida A. & Conrad M. Decoding ferroptosis for cancer therapy. Nat Rev Cancer, doi:10.1038/s41568-025-00864-1 (2025). 41073537

[90] Davis J. B. & Maher P. Protein kinase C activation inhibits glutamate-induced cytotoxicity in a neuronal cell line. Brain Res 652, 169–173, doi:10.1016/0006-8993(94)90334-4 (1994). 7953717

[91] Maher P. Potentiation of glutathione loss and nerve cell death by the transition metals iron and copper: Implications for age-related neurodegenerative diseases. Free Radic Biol Med 115, 92–104, doi:10.1016/j.freeradbiomed.2017.11.015 (2018). 29170091

[92] Quehenberger O. Lipidomics reveals a remarkable diversity of lipids in human plasma. J Lipid Res 51, 3299–3305, doi:10.1194/jlr.M009449 (2010). 20671299 PMC2952570

