# Supplementary material for: Acid ceramidase modulates the lipid profile and exacerbates sensitivity to ferroptosis in senescent cells
Source: Res Sq. 2025 Nov 28:rs.3.rs-8117957. Preprint. [Version 1] doi: 10.21203/rs.3.rs-8117957/v1 (PMC12676438; doi:10.21203/rs.3.rs-8117957/v1)
Supplement: 1 [file NIHPPRS8117957V1-supplement-1.pdf]

Supplemental Fig 1

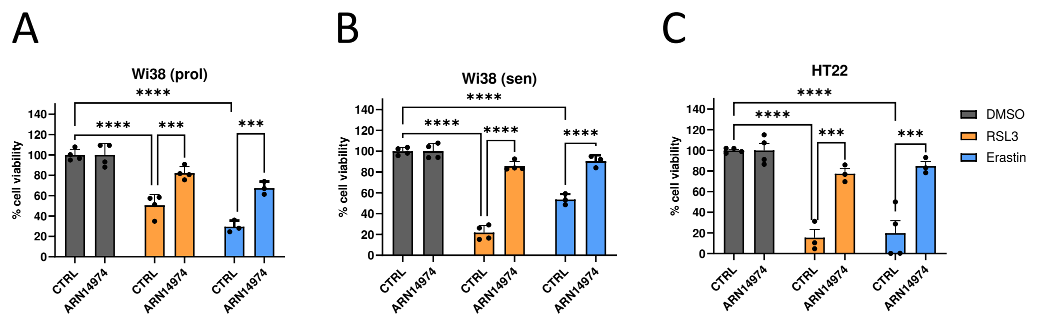

Figure S1. ACase specific chemical inhibitor ARN14974 protects both human Wi38 and murine HT22 cells. Percentage of cell survival of Wi38 proliferative (A) or senescent (B) cells and HT22 cells (C) against ferroptosis inducers RSL3 and erastin in the presence or absence of the ACase inhibitor ARN14974 (25 μM). ($^{**}p < 0.01$, $^{***}p < 0.001$, $^{****}p < 0.0001$. Two-way ANOVA). Values represent the mean ± SEM of at least 3 independent experiments. prol, proliferative; sen, senescent.

Supplemental Fig. 2

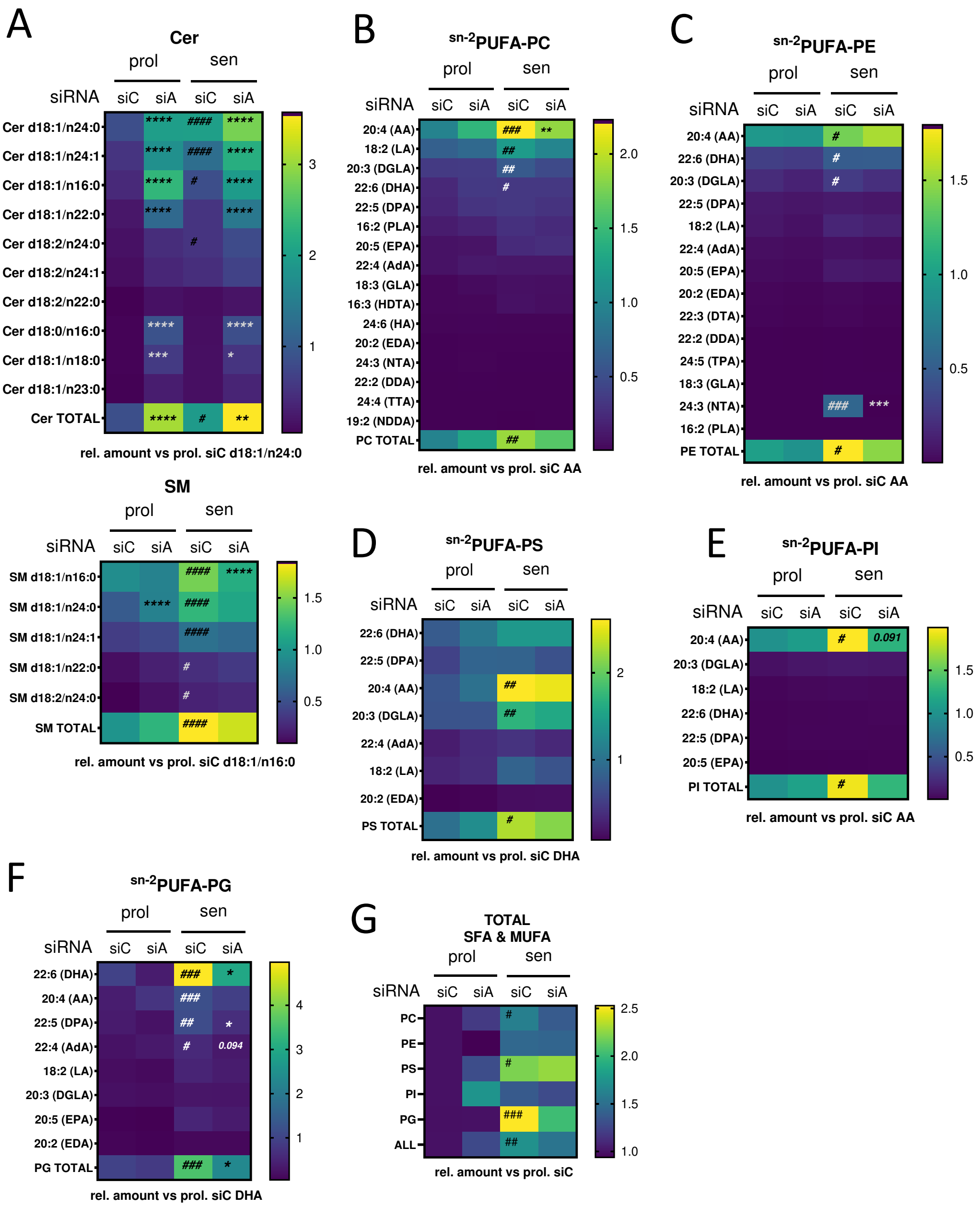

Figure S2. Heatmaps showing relative levels of the indicated lipid species compared to most abundant lipid in proliferative siCTRL cells. * indicates statistical significance between siCTRL and siACase in prol or sen cells. # indicates statistical significance between prol siCTRL and sen siCTRL. (*/#*p < 0.05*, **/##*p < 0.01*, ***/###*p < 0.001*, ****/####*p < 0.0001*. One-way ANOVA). prol, proliferative; sen, senescent; siC, CTRL siRNA; siA, ACase siRNA; Cer, ceramide; SM, sphingomyelin; SFA/MUFA, saturated fatty acids/monounsaturated fatty acids; PUFA, polyunsaturated fatty acid; AA, arachidonic acid; DHA, docosahexaenoic acid; DPA, docosapentanoic acid; DGLA, dihomo-gamma-linoleic acid; LA, linoleic acid; AdA, adrenic acid; EDA, eicosadienoic acid; PLA, palmitolinoleic acid; EPA, eicosapentaenoic acid; GLA, gamma-linoleic acid; HDTA, hexadecatrienoic acid; HA, herring acid; NTA, nervionic trienoic acid; DDA, docosadienoic acid; TTA, tetracosatetranoic acid; NDDA, nonadecatrienoic acid; PC, phosphatidylcholine; PE, phosphatidylethanolamine; PS, phosphatidylserine; PI, phosphatidylinositol; PG, phosphatidylglycerol.

Supplemental Fig. 3

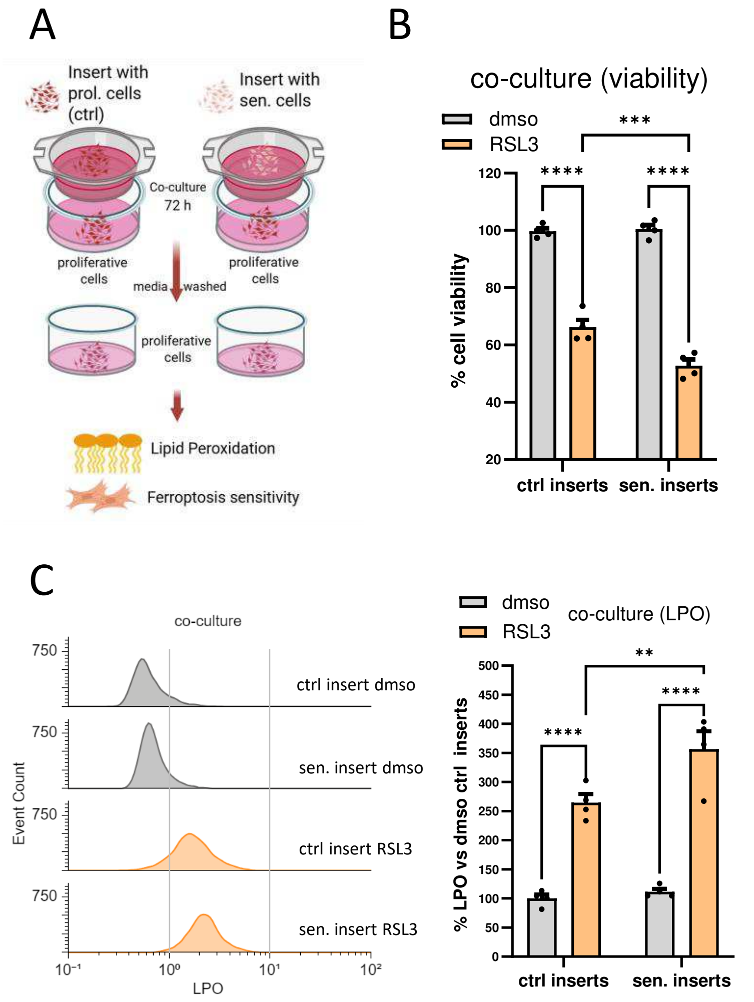

Figure S3. Co-culture with senescent cells sensitizes proliferative cells to ferroptosis. (A) Diagram illustrating the co-culture protocol. (B) Percentage of cell survival of proliferative cells after RSL3 treatment (250nM) in the presence or absence of senescent cells. (C) Representative flow cytometry histograms and quantification (bar graphs) showing LPO levels (C11-Bodipy 581/591) in proliferative cells after RSL3 treatment (250nM) in the presence or absence of senescent cells. ($^{**}p < 0.01$, $^{***}p < 0.001$, $^{****}p < 0.0001$. Two-way ANOVA). Values represent the mean ± SEM of at least 3 independent experiments. sen, senescent; LPO, lipid peroxidation.
